# Free posterior tibial flap reconstruction for hypopharyngeal squamous cell carcinoma

**DOI:** 10.1186/1477-7819-12-163

**Published:** 2014-05-24

**Authors:** Fei Chen, Jun Liu, Lihong Wang, Dan Lv, Yuanzhi Zhu, Qi Wu, Guojun Li, Hongliang Zheng, Xiaofeng Tao

**Affiliations:** 1 Department of Otolaryngology Head and Neck Surgery, West China Hospital, Sichuan University, 37 Guoxue Alley, Chengdu 610041, People’s Republic of China; 2 Department of Head and Neck Surgery, the University of Texas, MD Anderson Cancer Center, 1515 Holcombe Boulevard, Houston, TX 77030, USA; 3 Department of Otorhinolaryngology-Head and Neck Surgery, Changhai Hospital, Second Military Medical University, 168 Changhai Road, Shanghai 200433, People’s Republic of China; 4 Radiology Department of Shanghai Ninth People’s Hospital Affiliated Shanghai Jiaotong University School of Medicine, 639 Zhizaoju Road, Shanghai 200011, People’s Republic of China

**Keywords:** Hypopharyngeal cancer, Posterior tibial flap, Reconstruction

## Abstract

**Objectives:**

The aim of this article was to determine outcomes in patients with squamous cell carcinoma of the hypopharynx (SCCHP) in whom the free posterior tibial flap was used for primary reconstruction of hypopharynx defects after cancer resection.

**Subjects and methods:**

Between August 2009 and February 2012, 10 patients with SCCHP underwent posterior tibial flap reconstruction for hypopharynx defects. The corresponding clinical data were retrospectively collected and analyzed.

**Results:**

Despite the multistep and time-consuming procedure, the posterior tibial flap survival rate was 100%. Operation-induced complications did not occur in four patients. Six patients developed postoperative hypoproteinemia, four patients developed postoperative pulmonary infections, and four patients developed pharyngeal fistula. The pharyngeal and laryngeal functions of all patients were preserved.

**Conclusion:**

Our experience demonstrates that the posterior tibial flap is a safe and reliable choice for the reconstruction of hypopharynx defects.

## Background

Squamous cell carcinoma of the hypopharynx (SCCHP) is a highly aggressive malignant tumor and generally diagnosed at an advanced stage. SCCHP has the particular trait of invasiveness through the submucosa to induce distant lesions, also known as skip lesions [1], and thus total laryngopharyngectomy with adjuvant radiotherapy is widely utilized in late-stage SCCHP [2]. Although the majority of patients with selected pyriform sinus cancer treated with conservation surgery had successful laryngeal function preservation and better survival [3], pharyngolaryngeal reconstruction following SCCHP resection still remains a surgical challenge.

Over the years several pharyngolaryngeal reconstruction methods have been developed, such as the myocutaneous flap, gastric pull-up, and jejunal free flap techniques. With the development of microsurgical techniques, several fasciocutaneous free flaps from the radial forearm and anterolateral thigh have been widely utilized for the reconstruction of the hypopharynx [4]. Among the many free skin flaps available the radial forearm flap, which was first reported to be used for pharyngoesophageal reconstruction in 1985 [5], is the most popular free flap for head and neck soft tissue reconstruction after tumor extirpation. The radial forearm flap is the most popular fasciocutaneous flap for hypopharyngeal reconstruction, but its therapeutic benefits are accompanied by significant donor site morbidity, including a significant skin graft scar and wrist joint stiffness, which are proportional to the size of the harvested flap. It has been reported that if the septocutaneous perforators of the posterior tibia vessels are preserved the posterior tibial flap could be used in head and neck reconstruction after tumor removal [6]. The posterior tibial flap is also a fasciocutaneous flap and is located on the medial compartment of the leg with a blood supply coming from the septocutaneous perforators of vessels. Studies [7,8] have demonstrated that septocutaneous perforators of the posterior tibial artery are mainly distally concentrated on the medial surface of the leg and its vascular anatomy remains relatively constant. More and more evidence indicates that the posterior tibial flap can provide similar tissue properties but causes less morbidity than the radial forearm flap [6,7]. Due to these roles of the posterior tibial flapwe tried to begin using posterior tibial flap reconstruction for suitable patients with hypopharyngeal defects from August 2009.

In this article we present our initial clinical experience in utilizing the free posterior tibial flap for primary reconstruction of hypopharynx defects after tumor excision for the treatment of 10 SCCHP patients, and evaluate the procedure-related outcomes of these patients.

## Case presentation

The present retrospective case series analyzed the clinical and surgical data of 10 patients with SCCHP who underwent posterior tibial flap reconstruction for the treatmentof hypopharyngeal defects between August 2009 and February 2012 at the Department of Otolaryngology Head and Neck Surgery, West China Hospital of Sichuan University. Histopathologic diagnosis was conducted by the hospital’s pathology department. The TNM (Tumor, Lymph Node, Metastasis) classification was in accordance with UICC (Union for International Cancer Control) (2002).

All the patients underwent partial pharyngectomy and partial laryngectomy (Table 1). Two patients had partial cervical esophageal resection and another two patients had the removal of the involved thyroid gland. The tumors were completely removed and the resection margins were negative in all patients. For the seven patients without clinically palpable cervical lymph nodes, bilateral selective neck dissection was performed from levels II through IV. For the three patients with clinically palpable cervical nodes on the side of the primary tumor, modified radical neck dissection from levels II through V on the side of the primary lesion and selective neck dissection from levels II through IV on the contralateral side were performed. The size of the harvested posterior tibial flaps ranged from 5 × 7 cm to 7 × 12 cm, the average flap thickness was 1.2 cm (range, 0.9 to 1.4 cm), and the average pedicle length was 9 cm (range, 7 to 12 cm). During the microvascular anastomosis, the facial artery and facial vein were utilized in most of the patients (Table 2).

**Table 1:** Patient and flap data

**Case**	**Age**	**Sex**	**Tumor location**	**Tumor stage**	**Primary surgery**	**Neck lymph node dissection**	**Flap size (cm)**	**Flap thickness (cm)**	**Pedicle length (cm)**
1	42	M	PS	T3N2cM0	PPPL	Left:MRND Right:SND	7 × 10	1.4	12.0
2	67	M	PS	T3N0M0	PPPL	Left:SND Right:SND	6 × 9	1.4	7.0
3	47	M	PS	T3N1M0	PPPL	Left:SND Right:SND	6 × 9	1.3	11.0
4	63	M	PPW	T3N1M0	PPPL	Left:SND Right:SND	6 × 8	1.4	7.0
5	55	M	PS	T4N2cM0	PPEPL	Left:MRNDRight:SND	7 × 11	1.3	10.0
6	64	M	PPW	T3N1M0	PPPL	Left:SND Right:SND	5 × 7	0.9	7.0
7	47	M	PPW	T4N3M0	PPEPLTL	Left:SNDRight:MRND	7 × 12	1.0	12.0
8	53	M	PPW	T3N0M0	PPPL	Left:SND Right:SND	5 × 7	0.9	9.0
9	47	M	PPW	T4N2bM0	PPPLTL	Left:SND Right:SND	5 × 8	1.2	8.0
10	57	M	PPW	T3N1M0	PPPL	Left:SND Right:SND	5 × 7	1.1	7.0

MRND, Modified radical neck dissection; PPEPL, Partial pharyngoesophagectomy with partiallaryngectomy; PPEPLTL, Partial pharyngoesophagectomy with partiallaryngectomy and thyroid lobectomy; PPPL, Partial pharyngectomy with partial laryngectomy; PPPLTL, Partial pharyngectomy with partiallaryngectomy and thyroid lobectomy; PPW, Posterior pharyngeal wall; PS, Pyriformsinus; SND, Selective neck dissection.

**Table 2:** Anastomotic vessels

**Vessels**	**Numbers of cases**
**Arteries**	
Facial artery	6/10
Superior thyroid artery	4/10
**Veins**	
Common facial vein	8/20
Superior thyroid vein	6/20
Internal jugular vein	3/20
External jugular vein	3/20

All the patients received postoperative adjuvant radiotherapy. This retrospective review of medical records was approved by the Institutional Review Board of the West China Hospital (Cheng Du, Sichuan Province, China).

### Operative technique

Preoperative evaluation was conducted to identify patients who were suitable to undergo free posterior tibial flap reconstruction for hypopharynx defects. First, we excluded patients with severe signs and symptoms of peripheral artery disease of the lower limbs such as ischemic ulcers, intermittent claudication of the calf, and cold intolerance in the legs and feet. Second, an ultrasonic Doppler device (iU22 Philips, Beijing, People’s Republic of China) was utilized to show the position of the posterior tibial perforators and the direction of blood flow. In addition, when patients presented with varicose veins of the lower limbs on standing, they were referred for a lower limb venous evaluation before the operation because these patients were at high risk for deep vein thrombosis.

All patients underwent lymph node dissection. During the cervical lymph node dissection the available recipient vessels were carefully identified and preserved. Resection of the hypopharyngeal tumor with an adequate margin was carried out in three dimensions: the length of the resection margin was more than 25 mm and the depth of the margin reached the prevertebral fascia. Tumor-free margins were confirmed by intraoperative frozen sections.

**Figure 1:**
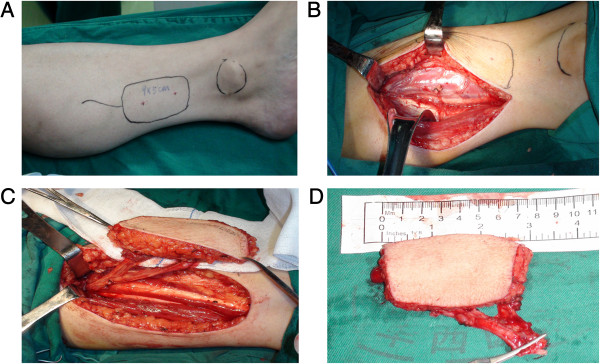
**The harvesting process of the free posterior tibial flap. A**: The posterior tibial flap was designed. **B**: The vessels of the posterior tibial were exposed. **C**: The posterior tibial flap was prepared. **D**: The posterior tibial flap was harvested.

**Figure 2:**
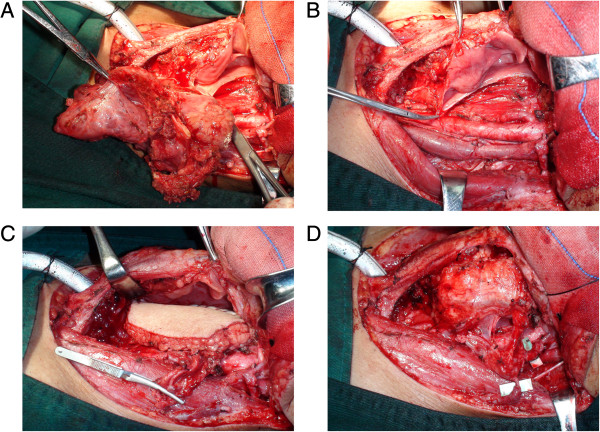
**The removal of SCCHP and the reconstruction for hypopharynx defects including the flap suture and microvascular anastomosis.** SCCHP, Squamous cell carcinoma of the hypopharynx. **A**: the hypoharyngeal tumor was resected completely. **B**: The recipient vessels were prepared for microvascular anastomosis. **C**: The flap suture was performed. **D**: The microvascular anastomosis was performed.

**Figure 3:**
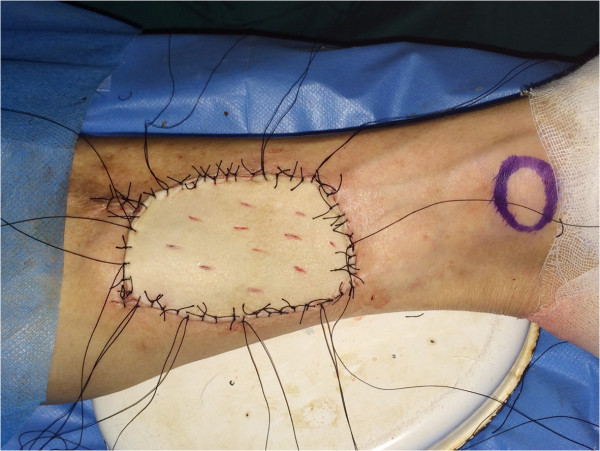
The skin graft was conducted on the leg donor site.

After the tumors were resected and the defects were measured, posterior tibial flaps were harvested according to the previous report (Figure 1) [6]. Once the recipient vessels were prepared the posterior tibial artery and veins were transected and the posterior tibial flap was transferred to the neck. As shown in Figure 2, the flap suture was performed first, followed by the microvascular anastomosis. Usually, one artery was anastomosed followed by two veins. The patency of the anastomosed vessels was evaluated by measuring skin temperature and monitoring the color of the transferred flap. A skin graft was conducted on the leg donor site (Figure 3).

### Postoperative treatment

Perioperative nutritional support, anticoagulant spasmolytic medications, anti-inflammatory drugs and dressing changes were utilized. The posterior tibial flap was also monitored postoperatively using electronic laryngoscopy. All the patients received postoperative adjuvant radiotherapy.

### Outcome evaluation

The following data were recorded: age, gender, primary tumor site, TNM classification, anastomosed vessels, treatment methods, the size of the flap, thickness of the flap, and pedicle length of the flap. The primary outcomes were the procedural complications, swallowing and speech functions, and patients’ post-treatment status (died or alive) during his or her period of respective follow-up.

### Patient demographics

The mean age of the patients was 54.2 years (range, 42 to 67 years), and all the patients were male (Table 1). All the patients were diagnosed with the primary SCCHP through histological examination. The primary tumor was located in the pyriform sinus in four patients and in the posterior pharyngeal wall in six patients. All patients had stage T3 or T4 tumors, and eightout of the ten patients had cervical nodal disease. None of the patients received any preoperative treatment.

### Surgical outcomes

**Figure 4:**
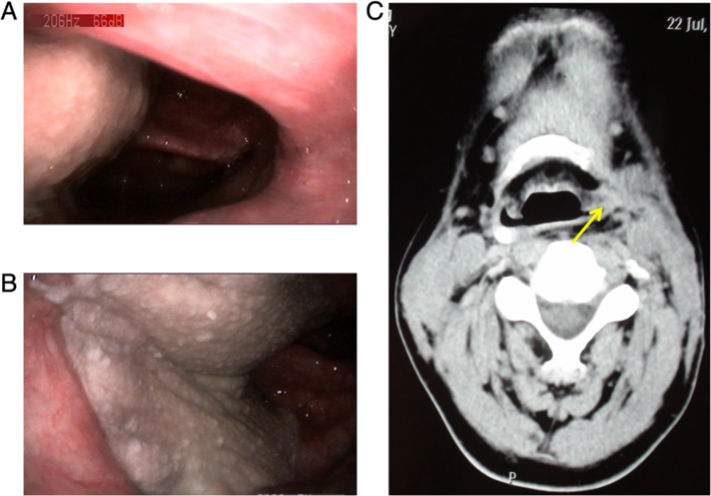
**Electronic laryngoscopy and computed tomography scan reveal the free posterior tibial flap and hypopharynx in a patient one year after reconstruction.** The yellow arrow indicates the posterior tibial flap. **A** and **B**: The posterior tibial flap and hypopharynx were revealed by electronic laryngoscopy through two different angles. **C**: The posterior tibial flap and hypopharynx were revealed by computed tomography.

The flap survival rate was 100%. Skin grafts at all the donor sites healed and the scars were painless. There were no donor site complications and no distal limb cold intolerance. All the patients were able to walk normally. Overall, four patients did very well, without any postoperative complications. However, six patients developed postoperative hypoproteinemia, 4 = four patients developed postoperative pulmonary infections, and four patients developed pharyngeal fistula. With various supporting treatments such as nutritional support, dressing changes, and systemic treatment with anti-inflammatory agents, all of these postoperative complications were resolved within four weeks. There were no late complications such as strictures (Figure 4).

### Functional outcomes

The average time to resumption of nasogastric feeding was seven days after the operation (range, 3 to 14 days), the swallowing function of all patients was preserved, and the average time to resumption of oral feeding was 33 days after the operation (range, 21 to 50 days). The laryngeal functions of all the patients were preserved and the patients were decannulated within a mean of 92.4 days postoperatively (range, 61 to 122 days).

### Oncologic outcomes

All the patients were followed up for at least 14 months (range, 14 to 42 months; mean, 31 months). One patient died of lung metastasis from SCCHP 24 months after the operation. Two patients had cervical lymph node metastasis 11 months and 12 months after the operation, respectively, and they received additional radical neck dissection. The remaining seven patients survived well without tumor relapse and metastasis at the time the data were collected for this study.

## Conclusions

Recently the posterior tibial flap has received considerable interest for its use in the reconstruction of distal sites [6,9,10]. In this study we investigated the outcomes of patients for whom the posterior tibial flap was used for primary reconstruction of hypopharynx defects after SCCHP extirpation. We found that the posterior tibial flap has many advantages in the reconstruction of the pharynx and hypopharynx. Firstly, as reported in many anatomic studies [11,12], little variation was observed in the vascular anatomy of the lower limb. The septocutaneous perforators always run on the deep transverse fascia of the leg in the septum between the flexor digitorumlongus and soleus, and a large number of these septocutaneous perforators could be found at the distal leg. Therefore, compared with other fasciocutaneous flaps, the harvest of the posterior tibial flap is much simpler. Secondly, our study showed that the mean thickness of the skin paddle was 1.2 cm and the size of the flaps ranged from 5 × 7 cm to 7 × 12 cm. The posterior tibial flap is a thin and pliable flap.

Compared with the anterior lateral thigh flap, the posterior tibial flap is particularly suitable for resurfacing defects of the hypopharyngeal mucosa with optimal functional and aesthetic outcomes, although the anterior lateral thigh flap does not require skin graft on the leg donor site for almost all cases. Lastly, since these flaps provide a long pedicle and large vessels, the microvascular anastomosis is presumably safe and the choice of recipient vessels in the neck is flexible, which is especially important for patients with a history of previous surgery or radiotherapy.

However posterior tibial flap reconstruction has its limitations. A major tributary of the lower limb is sacrificed which might jeopardize circulation and subsequently induce ischemia. Although the distal leg is supplied with redundant blood flow and multiple arterial-arterial connections that provide a rich vascular network even after removal of the posterior tibial artery [13], strict selection criteria should be used to exclude patients with insufficient distal leg circulation. By following such criteria, we ensured that no worsening of peripheral vascular disease or distal limb ischemia occurred in the present study during the postoperative period.

Posterior tibial flap reconstruction also may complicate wound healing and cause hemorrhage, necrosis, pulmonary infections, and fistula. In our studyfour patients developed pharyngeal fistulas which were accompanied by hypoproteinemia which might be associated with the patients’ severe nutritional deficiencies. Well-vascularized coverage of the reconstructed hypopharynx, exposed vessels of the operating field, and surrounding cutaneous defect can help avoid these complications. In addition, perioperative nutritional support and dressing changes are very important to prevent complications, and anti-inflammatory drugs should also be considered.

Optimal reconstructive procedures should cause the least mortality and morbidity and the best preservation of the functions of the hypopharynx and larynx [14]. In the current study, the posterior tibial flaps survived and the hypopharyngeal and laryngeal functions were preserved in all the patients, resulting in good quality of life for the patients. The most important factor in the survival of posterior tibial flaps is to keep the patency of the anastomosed vessels. We found that four factors in particular contributed to the patency of the anastomoses and the survival of the posterior tibial flaps: (1) when the calibers of the donor vein and acceptor vein were significantly different, the two veins were joined with an end-to-side anastomosis (for example, the end of the posterior tibial vein was anastomosed with the side of the internal jugular vein) to prevent possible venous thrombosis through the negative pressure of the jugular vein; (2) Two pairs of veins were usually anastomosed to ensure adequate blood backflow and consequently reduce the congestion and edema of the flaps; (3) postoperative anticoagulant spasmolytic medications were applied; and (4) the posterior tibial flap was frequently monitored postoperatively using electronic laryngoscopy to identify any early disruption to the blood supply.

It has been reported that there is a high incidence of submucosal tumor extension along the longitudinal axis of the hypopharynx in patients with SCCHP and that these tumors extend from 10 to 25 mm into the submucosa [15,16]. Apparently, the complete resection of tumor is another important factor affecting the outcomes of reconstruction. Therefore, in our study we cautiously adhered to appropriate resection margins in three dimensions during surgery for SCCHP to ensure sufficient safe margins.

To date, the management of lymph nodes in hypopharyngeal cancer remains a challenging problem. It has been reported that for patients with clinically negative cervical lymph nodes, 36% of cervical lymph nodes on the side of the primary tumor and 27% of contralateral cervical lymph nodes contained a metastatic tumor [17]. Because of the high propensity for early metastasis to the contralateral neck, selective neck dissection on the contralateral cervical lymph nodes was carried out for patients with positive or negative cervical lymph nodes on the side of the primary tumor and followed by postoperative radiotherapy, which is effective for the treatment of lymph node metastases.

In the present study we obtained satisfactory results using posterior tibial flaps, including few postoperative complications, short time to feeding, functional organ preservation in the larynx and hypopharynx, and acceptable quality of life. However, a longer follow-up period is required to investigate the actual survival rate for future survival rate evaluation. In summary, our experience demonstrates that the posterior tibial flap is a safe and reliable choice for the reconstruction of hypopharynx defects.

## Consent

Written informed consent was obtained from all patients for presentation of the paper and accompanying images.

## Abbreviations

SCCHP: Squamous cell carcinoma of the hypopharynx; TNM: Tumor, Lymph Node, Metastasis; UICC: Union for International Cancer Control.

## Competing interests

The authors declare that they have no competing interests.

## Authors’ contributions

Study conception and design: LG, TX, CF, LJ. Acquisition of data: CF, LJ, WL, LD, ZY, WQ. Data Analysis and interpretation: LG, TX, CF, LJ, WL, LD, ZY, WQ. Drafting of manuscript: LG, TX, CF, LJ, WL, LD, ZY, WQ. Critical revision: LG, TX, CF, LJ. All authors read and approved the final manuscript. All authors read and approved the final manuscript
